# Awareness, utilization, and implementation barriers of the wheelchair skills program and test in Korea: a survey of users and clinicians

**DOI:** 10.3389/fpubh.2025.1711971

**Published:** 2026-01-14

**Authors:** Nayoung Jeong, Doyeol Kim, Seonhong Hwang

**Affiliations:** 1 Department of Physical Therapy, Graduate School of Hoseo University, Asan-si, Chungcheongnam-do, Republic of Korea; 2 Research Institute for Basic Sciences, Hoseo University, Asan-si, Chungcheongnam-do, Republic of Korea; 3 Smart Healthcare Convergence Research Center, Hoseo University, Asan-si, Chungcheongnam-do, Republic of Korea

**Keywords:** wheelchair skills program (WSP), wheelchair skills test (WST), wheelchair users, rehabilitation professionals, awareness, implementation barriers, South Korea

## Abstract

**Background:**

The Wheelchair Skills Program (WSP) and Wheelchair Skills Test (WST) have been widely validated and implemented in Western rehabilitation settings. However, although the WSP and WST have been introduced in Korea, the extent of their awareness and use remains unclear. In addition, their clinical- and community-level integration has not yet been systematically evaluated.

**Objective:**

To investigate the awareness, utilization, and perceived necessity of WSP and WST among wheelchair users and clinical professionals in Korea, and to identify barriers to implementation and preferences for future program development.

**Methods:**

A nationwide cross-sectional survey was conducted between August 2023 and July 2024. A total of 134 wheelchair users and 120 clinical professionals (physical therapists) participated. Validated questionnaires assessed demographics, wheelchair skill usage and training experience, awareness of WSP/WST, and preferences for program formats.

**Results:**

Awareness of WSP and WST was critically low in both groups: 98.3% of clinicians and 82.1% of users reported no knowledge of related training manuals or tools. Only 5.0% of clinicians had ever used training or assessment materials. Among clinicians, the most frequently taught skills were transfer (65%), body lift (48.3%), and forward propulsion (35%). In contrast, users most frequently performed forward propulsion (47.8%), reverse propulsion (34.3%), and turning (30.6%), highlighting a mismatch between instruction and real-world demands. Over 70% of respondents in both groups agreed that a Korean-style wheelchair skills program would be useful (71.7% of clinicians; 89.6% of users). Preferred formats differed: 56.7% of clinicians favored mobile/online delivery, while users expressed similar interest in both mobile platforms (38.3%) and in-person training by professionals (35.8%). Barriers to implementation included high patient loads (47.8% treating over 20 patients daily) and a national shortage of physical therapists (0.84 per 1,000 population, below the OECD average of 1.09).

**Conclusion:**

Despite the introduction of K-WSP, awareness and utilization of wheelchair skill training programs remain extremely limited in Korea. There are clear gaps between clinical practice and user needs, as well as structural barriers such as workforce limitations and lack of institutional support. To address these issues, national strategies must promote curricular integration, continuing education, policy-level support, and digital platform development to enable scalable wheelchair skill education.

## 1 Introduction

According to the World Health Organization (WHO), approximately 1.3 billion people, or 16% of the global population, experience significant disabilities, and this number is expected to rise due to aging populations and increased prevalence of chronic diseases (1). In the European Union (EU), more than 26.8% of individuals aged 16 and over reported having a disability in 2022 (2). In the United States, about 13% of the population had a disability in 2024, with approximately 3.6 million individuals using wheelchairs (3). In China, recent data indicates that approximately 43.2% of older adults with chronic conditions live with a disability (4), while India’s estimates of the total disabled population range between 50 and 80 million individuals (5).

The Wheelchair Skills Program (WSP) is a program developed to enhance wheelchair skills for safe and efficient mobility of wheelchair users (6, 7) and the Wheelchair Skills Test (WST) is a tool for measuring the wheelchair using ability of wheelchair users (8, 9). According to previous studies, WSP participants have shown improved activity participation and wheelchair operating skills in daily life (7, 8, 10–13), and the combination of WST and individualized skills training programs has been shown to significantly improve wheelchair operating skills of wheelchair users, leading to improved safety, balance, and pulmonary function (14–16).

In Europe and North America, WSP and WST are widely incorporated into rehabilitation programs and community-based training initiatives (7, 12, 17–19). However, in Asia, the utilization of WSP and WST remains relatively unclear. Although Japan and China have advanced rehabilitation programs and large wheelchair user populations, there is limited evidence in the international literature regarding systematic implementation or large-scale research on WSP or WST in these countries. This suggests a potential gap in structured wheelchair skill training across parts of Asia.

In Korea, a Korean-adapted manual Wheelchair Skills Training Program for individuals with hemiplegia (K-WSTP) was developed and evaluated in 2016. In the Wheelchair Skills Program/Test, manual wheelchair skills comprise 34 items and are classified into three difficulty levels: basic, intermediate, and advanced. Based on this framework, the Korean program selected 11 basic-level skills and tailored the training content for individuals with hemiplegia (20).

However, despite its introduction, there is limited data on the actual dissemination and utilization of K-WSP in clinical settings or community rehabilitation centers in Korea. Unlike Europe and North America, where WSP and WST are systematically integrated into physical therapy and occupational therapy education, K-WSP’s integration into Korean rehabilitation education remains limited. Furthermore, there are no large-scale studies or government-published statistics that reflect the awareness and practical application of WSP or WST among Korean physical therapists, occupational therapists, or wheelchair users. This lack of structured data makes it challenging to evaluate the current status and effectiveness of K-WSP in improving wheelchair skills. Moreover, policy initiatives for standardized wheelchair skills training remain underdeveloped, further limiting access to structured programs.

Although WSP and WST have been shown to improve wheelchair mobility and safety in Western contexts, their application and effectiveness in Korea remain underexplored. The lack of systematic training programs for wheelchair skills in clinical education, combined with limited policy support for community-based wheelchair skills training, limits the opportunity for effective skill development and independence for wheelchair users. Moreover, Korean rehabilitation programs tend to focus more on physical recovery and less on practical mobility training, further widening the gap in wheelchair skill proficiency.

The absence of national-level data on WSP/WST utilization in clinical and community settings indicates a critical research gap. Without reliable data, it is challenging to assess the effectiveness of K-WSP or plan for its broader implementation in rehabilitation settings. It remains unclear how many Korean wheelchair users or rehabilitation professionals are aware of WSP/WST or have access to structured training. Additionally, while K-WSP has been introduced, its practical application and real-world impact are not well-documented.

This study aims to identify the current awareness and utilization status of WSP and WST among wheelchair users and clinical professionals in Korea, and to investigate the necessity and preferred methods for the development and dissemination of an improved Korean version of WSP and WST. Specifically, the study seeks to assess the awareness and utilization of WSP, WST, and K-WSP in Korean clinical and community settings, while also identifying the barriers that prevent the effective implementation of wheelchair skills training programs. Furthermore, it explores the educational needs and policy requirements necessary for the successful integration of K-WSP into Korean rehabilitation practices. Based on these objectives, the following hypotheses are proposed: (Hypothesis 1) The awareness and utilization of WSP and WST are significantly lower in Korea compared to Western countries. (Hypothesis 2) Clinical professionals in Korea are less likely to integrate wheelchair skills training into their rehabilitation programs due to a lack of structured education and policy support. (Hypothesis 3) Wheelchair users in Korea express a need for improved skill training programs and better community-based accessibility. The findings from this study are expected to provide foundational data for policy development and the strategic dissemination of K-WSP in Korea.

## 2 Methods

### 2.1 Survey development and dissemination

For this study, separate questionnaires were developed for wheelchair users and clinical experts. The questionnaire for wheelchair users consisted of a section asking about demographic and basic medical information such as gender, age, height, weight, diagnosis, injury level, functional level, and rehabilitation therapy experience (11 questions); a section about wheelchair usage history, including the type of wheelchair being used, the duration of use, daily usage hours, and a self-evaluation of wheelchair proficiency (4 questions); and a section related to wheelchair operation skills, including frequently performed wheelchair operation techniques, experiences with education, training, and evaluation of wheelchair operation techniques, and the usefulness, willingness to participate, and preferred types of a Korean wheelchair operation skills training and evaluation program (20 questions).

The questionnaire for clinical experts consisted of two sections: a section asking about demographic and career information excluding the wheelchair usage history section (13 questions), and a section asking about the Korean wheelchair operation skills training and evaluation program (16 questions). In addition to demographic information, clinical experts were asked about their occupation, clinical experience, type of employing institution, location, number of patients in charge, and the number of patients treated per day, as well as reasons for difficulty in participating in the Korean wheelchair operation skills training and evaluation program.

The survey was conducted in four stages: the first stage from August 24, 2023 to October 29, 2023; the second stage from October 30, 2023 to February 18, 2024; the third stage from February 19, 2024 to March 3, 2024; and the fourth stage from June 1, 2024 to July 30, 2024. Assistance for the survey was provided by the Korea Spinal Cord Injury Association, the Korea Spinal Cord Injury Gyeonggi-do Association, the Korea Spinal Cord Injury Chungcheongnam-do Association, Chungcheongnam-do Office, the Korean Disabled Rugby Association, the Korean Disabled Basketball Federation, the Korean Disabled Table Tennis Association, the Korean Disabled Curling Association, the Korean Physical Therapist Association, the Gyeonggi-do and Chungcheongnam-do branches of the Korean Physical Therapist Association, and the Naver Club of the Korean Physical Therapists.

The survey items were developed and validated through a multi-stage process from 2022 to 2023. Initially, a panel of experts, including physical therapists, rehabilitation medicine specialists, and wheelchair users, reviewed the survey items to evaluate their relevance and clarity. Following this, a pilot test was conducted with 15 clinical experts (physical therapists and occupational therapists) and 15 wheelchair users, totaling 30 participants. The purpose of the pilot test was to assess the understandability and appropriateness of the questions across both groups. Feedback from the pilot test was used to refine the survey items for better clarity and relevance. Based on the expert evaluations, the Content Validity Index (CVI) was calculated for each item, and only items with a CVI of 0.8 or higher were included in the final survey. This step ensured that the questions were both relevant and clear for the targeted population.

To evaluate the internal consistency of the survey, Cronbach’s Alpha was calculated for each section. The results showed high reliability across all sections, with Demographic Information scoring 0.81, Wheelchair Usage History scoring 0.84, Wheelchair Skill Proficiency scoring 0.83, Clinical Professional Information scoring 0.79, and Skill Training and Evaluation scoring 0.82. All values exceeded the commonly accepted threshold of 0.7, indicating strong internal consistency and reliability of the survey items.

This study was approved by the Institutional Review Board (IRB) of Hoseo University (1041231-180103-HR-068-03). Participants received an explanation about the study and signed a consent form before receiving the questionnaire. They agreed to the collection and use of information through the survey before responding to the questionnaire. To avoid duplicate responses, names, addresses, email addresses, and license numbers (for clinical experts) were collected, but all were discarded after checking for duplicates, and the data were anonymized.

### 2.2 Statistical analysis

Descriptive statistics were used to illustrate the demographic characteristics and survey responses of both wheelchair users and clinical experts. These included frequencies, percentages, means, and standard deviations, which were calculated to provide an overview of the participants’ profiles and responses to key survey items.

To compare the responses between wheelchair users and clinical experts, Chi-square tests were employed for categorical variables, such as awareness of wheelchair skills programs, participation in training sessions, and perceived proficiency in wheelchair skills. For continuous variables, such as the duration of wheelchair use and daily usage hours, independent *t*-tests were conducted to determine group differences. Statistical significance was set at *p* < 0.05 with a two-tailed test.

The internal consistency of the survey items was assessed using Cronbach’s Alpha for each section of the questionnaire, as mentioned in Section 2.1. All sections demonstrated acceptable reliability with values exceeding 0.7, indicating strong internal consistency across survey items.

All survey responses were first organized and pre-processed using Microsoft Excel (Microsoft Corporation, Redmond, Washington, DC, USA), and then analyzed with IBM SPSS Statistics version 24 (IBM Corp., Armonk, NY, USA). For visualization of key findings, bar graphs and pie charts were generated to depict the distribution of responses for major survey items.

## 3 Results

### 3.1 Respondents

A total of 134 wheelchair users and 120 clinical experts voluntarily participated in this study. All participants provided informed consent prior to survey participation, and their personal information was anonymized to ensure confidentiality. The selection criteria for wheelchair users included those who had been using a wheelchair for 6 months or more and were able to use electronic devices for survey completion. Participants who had been using a wheelchair for less than 6 months or were unable to operate electronic devices were excluded from the study. For clinical experts, the selection criteria required experience treating manual or electric wheelchair users, specifically in the roles of physical therapists or occupational therapists. Those who had no experience with wheelchair users were excluded from the survey.

#### 3.1.1 Results for clinical professionals

The response results for personal information, workplace, and workplace information are shown in Table 1. The study participants consisted of 32 men (26.6%) and 88 women (73.3%), with more women than men. All respondents were physical therapists by occupation, and their ages were as follows: 92 people (76.6%) in their 20s, 18 people (15%) in their 30s, 8 people (6.6%) in their 40s, and 2 person (1.6%) in their 50s. Clinical experience was as follows: less than 6 months for 5 people (4.2%), 7 months to 1 year for 34 people (28.3%), 1–3 years for 35 people (29.2%), 4–6 years for 24 people (20.0%), 7–9 years for 9 people (7.5%), and 9–20 years for 13 people (10.8%). The types of workplaces were as follows: hospitals (with more than 30 beds) had the most respondents at 56 (46.7%), followed by general hospitals (with more than 100 beds) at 34 (28.3%), clinics (with less than 30 beds) at 18 (15%), nursing hospitals at 10 (8.3%), and community centers or nursing homes at 2 (1.7%). This classification reflects the diversity of clinical settings where wheelchair skills training might be applied. According to the standards of the National Health Insurance Corporation, there were 74 respondents (61.7%) from secondary hospitals, 32 (27.7%) from primary hospitals, 6 (5.0%) from tertiary hospitals, and 8 (6.7%) from etc. The number of therapists at the workplace was as follows: 50 people (41.7%) had 11–15 colleagues, 14 people (11.7%) had 16–20 colleagues, 16 people (13.3%) had more than 31 colleagues, 20 people (16.7%) had 6–10 colleagues, 12 people (10%) had 5 or fewer colleagues, and 8 people (6.7%) had 21–30 colleagues.

**Table 1:** Demographic and workplace characteristics of clinical professionals (*N* = 120).

Division	Contents	Frequency (*N*, %)
Sex	Male	32 (26.6)
	Female	88 (73.3)
Type of job	Physical therapist	120 (100)
Age (year)	20–29	92 (76.6)
	30–39	18 (15)
	40–49	8 (6.6)
	50–59	2 (1.6)
Clinical experience (years)	Less than 6 months	5 (4.2)
	7 months–1 year	34 (28.3)
	1–3	35 (29.2)
	4–6	24 (20.0)
	7–9	9 (7.5)
	9–20	13 (10.8)
Type of workplace	Community center or nursing home	2 (1.7)
	Nursing hospital	10 (8.3)
	Clinic	18 (15.0)
	Hospital	56 (46.7)
	General hospital	34 (28.3)
Number of therapists	Less than 5	12 (10.0)
	6–10	20 (16.7)
	11–15	50 (41.7)
	16–20	14 (11.7)
	21–30	8 (6.7)
	More than 31	16 (13.3)

#### 3.1.2 Number of patients in charge

The number of patients currently under care was distributed as follows: 1–20 patients for 76 people (63.3%), 20–40 patients for 22 people (18.3%), 40–60 patients for 10 people (8.3%), and 60 or more for 12 people (10%). The number of patients treated per day was 10–20 patients for 76 people (63.3%), 20–40 patients for 24 people (20%), and 40–150 patients for 20 people (16.7%). The average number of patients treated per day in the department was 1–75 patients for 72 people (58.8%), 76–150 patients for 36 people (31.36%), 151–225 patients for 8 people (7.84%), and 226–300 patients for 4 people (3.92%). The number of wheelchair users treated per day was 0–10 patients for 82 people (68.3%), 11–20 patients for 24 people (20%), 21–30 patients for 6 people (5%), 31–40 patients for 2 people (1.7%), and 6 people (5%) reported they did not know (Table 2).

**Table 2:** Distribution of patients and wheelchair users in clinical settings (*N* = 120).

Division	Contents	Frequency (*N*, %)
Current patient caseload	1–20	76 (63.3)
	20–40	22 (18.3)
	40–60	10 (8.3)
	More than 60	12 (10.0)
Number of patients treated by oneself per day	10–20	76 (63.3)
	20–40	24 (20.0)
	40–150	20 (16.6)
Number of patients treated by department per day	1–75	72 (60.0)
	76–150	36 (30.0)
	151–225	8 (6.6)
	226–300	4 (3.4)
Number of wheelchair users treated per day	0–10	82 (68.3)
	11–20	24 (20.0)
	21–30	6 (5.0)
	31–40	2 (1.6)
	Not known	6 (5.0)

#### 3.1.3 Wheelchair skill education

As shown in Table 3, when asked about the most frequently taught wheelchair skills to patients, 78 respondents (65%) selected “transfer,” followed by “lifting the body from the seat” (58 respondents, 48.3%) and “forward propulsion” (42 respondents, 35%). Regarding the three most important wheelchair skills for independent use—excluding those commonly taught in clinical settings—54 respondents (45.3%) included “lifting the body from the seat” among their top choices, while “transfer” and “forward propulsion” were each selected by 40 respondents (33.3%). Other skills such as “backward propulsion” and “ramp up/down” were each selected by 2 respondents (1.7%), and 8 respondents (6.7%) mentioned skills such as “footrest adjustment,” “braking,” or provided no specific preference. These findings indicate that while transfer training is most frequently emphasized in clinical settings, respondents consider lifting from the seat to be the most critical skill for users’ independence (Table 4).

**Table 3:** Frequency of the most frequently taught and the most important wheelchair skills according to respondents (*N* = 120).

Contents		Frequency (*N*, %)
The most frequently taught wheelchair skills to patients	Transfer	78 (65.0)
	Body lift	58 (48.3)
	Forward propulsion	42 (35.0)
The most important skills for wheelchair users	Body lift	54 (45.3)
	Transfer	40 (33.3)
	Forward propulsion	40 (33.3)

**Table 4:** Experience in wheelchair skill training and evaluation (*N* = 120).

Division	Contents	Frequency (*N*, %)
Learned wheelchair skills from an expert	Yes	50 (41.7)
	No	70 (58.3)
Types of professional educators	Professor	16 (32.0)
	Peer therapist	13 (26.0)
	Instructor of qualification training program	6 (12.0)
	etc. (self, online platforms)	2 (4.0)
	Do not know	13 (26.0)
Experience in using tools for training and evaluating wheelchair skills	Yes	6 (5.0)
	No	114 (95.0)
Tools for training wheelchair skills	None	114 (95.0)
	Books	4 (3.3)
	Skateboard or sling	2 (1.7)
Knowledge of manuals to train and evaluate wheelchair skills	Know	2 (1.7)
	Do not know	118 (98.3)
Knowledge of WST-Q	No	118 (98.3)
	Yes	2 (1.7)

#### 3.1.4 Experience in wheelchair skill training and evaluation

Among the 120 respondents, 50 individuals (41.7%) reported having received wheelchair skill training from an expert, while 70 individuals (58.3%) had not. Regarding the types of educators, 16 respondents (32.0%) identified professors, 13 (26.0%) reported peer therapists, 6 (12.0%) indicated instructors from qualification training programs, and 2 (4.0%) mentioned other sources such as self-learning or online platforms. Additionally, 13 individuals (26.0%) answered “Do not know” when asked about the educator. Only 6 respondents (5.0%) reported experience using tools for training and evaluating wheelchair skills, while 114 (95.0%) did not. Regarding the types of tools used, 114 respondents (95.0%) reported using none, whereas 4 (3.3%) used books and 2 (1.7%) used alternative tools such as skateboards or slings. Awareness of training and evaluation manuals was also low, with only 2 respondents (1.7%) indicating they were aware of such manuals, and 118 (98.3%) reporting no knowledge. Similarly, only 2 respondents (1.7%) reported being familiar with the Wheelchair Skills Test Questionnaire (WST-Q), while the remaining 118 (98.3%) were not aware of it.

#### 3.1.5 Perceived usefulness of disseminating the Korean version of the wheelchair skill program and willingness to participate in education programs

The perceived usefulness of disseminating the Korean version of the wheelchair skill program, which includes both training and testing tools, is summarized in Table 5. Among the 120 respondents, 36 individuals (30.0%) rated it as very useful, 50 (41.7%) as useful, 30 (25.0%) as neutral, and 4 (3.3%) as useless. No respondents selected “not at all useful.” Regarding the level of agreement with the statement, “Long-term wheelchair users may have improper techniques,” 12 respondents (10.0%) strongly agreed, 68 (56.7%) agreed, 26 (21.7%) were neutral, and 14 (11.7%) disagreed. No respondents strongly disagreed. When asked about their willingness to participate in training or education programs related to wheelchair skills, 78 respondents (65.0%) answered “yes,” and 42 (35.0%) answered “no.” As for preferred methods of participation, 68 respondents (56.7%) favored online or mobile applications, 30 (25.0%) preferred professional training courses, 14 (11.7%) selected self-learning through materials such as books, papers, and manuals, and 8 (6.7%) preferred participation in education and research programs at schools or research institutes. Reported barriers to participation included difficulty in making time (49 respondents, 40.7%), perceiving the program as unnecessary (28 respondents, 23.7%), cost issues (26 respondents, 22.0%), trust issues (8 respondents, 6.8%), and other reasons (8 respondents, 6.8%). In terms of the usefulness of digital tools for clinical professionals, 22 respondents (18.3%) found them very useful, 62 (51.7%) found them useful, and 36 (30.0%) were neutral. None rated them as useless or not at all useful. Similarly, regarding the usefulness of such tools for wheelchair users, 46 respondents (38.3%) considered them very useful, 54 (45.0%) considered them useful, and 20 (16.7%) were neutral. Again, no respondents rated them as useless or not at all useful (Table 5).

**Table 5:** Perceived usefulness of disseminating the Korean version of the wheelchair skill program for clinicians/users (*N* = 120).

Division	Contents	Frequency (*N*, %)
Perceived usefulness of disseminating the Korean version of the wheelchair skill program	Very useful	36 (30.0)
	Useful	50 (41.7)
	Neutral	30 (25.0)
	Useless	4 (3.3)
	Not at all useful	0 (0)
Agreement with: “Long-term wheelchair users may have improper techniques”	Strongly agree	12 (10.0)
	Agree	68 (56.7)
	Neural	26 (21.7)
	Disagree	14 (11.7)
	Strongly disagree	0 (0)
Willingness to participate in training/education programs	Yes	78 (65.0)
	No	42 (35.0)
Preferred method of participation	Professional training course	30 (24.9)
	Self-learning through documents such as books, papers, and manuals	14 (11.7)
	Website or mobile application	68 (56.7)
	Participating in education and research at schools or research institutes	8 (6.7)
Barriers to participation	Difficulty in making time	49 (40.7)
	Unnecessariness	28 (23.7)
	Cost issue	26 (22.0)
	Trust issue	8 (6.8)
	others	8 (6.8)
Usefulness of digital tools (clinical professionals)	Very useful	22 (18.3)
	Useful	62 (51.7)
	Neutral	36 (30.0)
	Useless	0 (0)
	Not at all useful	0 (0)
Usefulness of digital tools (wheelchair users)	Very useful	46 (38.3)
	Useful	54 (45.0)
	Neutral	20 (16.7)
	Useless	0 (0)
	Not at all useful	0 (0)

### 3.2 Results for wheelchair users

#### 3.2.1 Respondents

A total of 134 wheelchair users participated in the survey, of whom 114 (85.1%) were male and 20 (14.9%) were female. The most common age groups were 40–49 years and 50–59 years, each comprising 42 respondents (31.3%). This was followed by 30 respondents (22.4%) aged 60–69 years, 20 (14.9%) aged 30–39 years, 10 (7.5%) aged 20–29 years, and 8 (6.0%) aged 10–19 years. In terms of height, 60 respondents (44.8%) were 170–179 cm tall, 36 (26.9%) were taller than 180 cm, 32 (23.9%) were 160–169 cm, and 6 (4.8%) were 150–159 cm. Regarding body weight, 40 respondents (29.8%) weighed 70–79 kg, followed by 34 (25.4%) who weighed 80–89 kg, 30 (22.4%) who weighed 60–69 kg, 16 (11.9%) who weighed 50–59 kg, 8 (6.0%) who weighed 40–49 kg, 4 (3.0%) who weighed more than 100 kg, and 2 (1.5%) who weighed 90–99 kg. With respect to diagnosis, the majority (120 respondents, 89.6%) had spinal cord injury (SCI), while 2 (1.5%) reported polio and 12 (9.0%) reported other conditions. As for the level of injury, 78 respondents (58.2%) had thoracic injuries, 46 (34.3%) had cervical injuries, and 10 (7.5%) had lumbar injuries. Functional level based on the ASIA scale was reported as Grade A in 28 respondents (20.9%), Grade B in 8 (6.0%), Grade C in 6 (4.5%), and Grade D in 4 (3.0%). A substantial proportion of respondents (88 individuals, 65.7%) reported that they did not know their ASIA classification (Table 6).

**Table 6:** Personal information of wheelchair users (*N* = 134).

Division	Contents	Frequency (*N*, %)
Gender	Male	114 (85.1)
	Female	20 (14.9)
Age (year)	10–19	8 (6.0)
	20–29	10 (7.5)
	30–39	20 (14.9)
	40–49	42 (31.3)
	50–59	42 (31.3)
	60–69	12 (9.0)
Height (cm)	150–159	6 (4.8)
	160–169	32 (23.9)
	170–170	60 (44.8)
	More than 180	36 (26.9)
Weight (kg)	40–49	8 (6.0)
	50–59	16 (11.9)
	60–69	30 (22.4)
	70–79	40 (29.8)
	80–89	34 (25.4)
	90–99	2 (1.5)
	More than 100	4 (3.0)
Diagnosis	SCI	120 (89.6)
	polio	2 (1.5)
	Etc.	12 (9.0)
Level of injury	Cervical	46 (34.3)
	Thoracic	78 (58.2)
	Lumbar	10 (7.5)
Functional level (ASIA impairment scale)	A	28 (20.9)
	B	8 (6.0)
	C	6 (4.5)
	D	4 (3.0)
	Unknown	88 (65.7)

#### 3.2.2 Rehabilitation therapy experience

The number of medical institutions where treatment was received showed that 102 people (76.1%) had been treated at 5 or fewer institutions, and 32 people (23.9%) at 10 or fewer institutions. The number of all therapists who had treated them was the highest with 62 people (46.3%) having been treated by 10–19 therapists, followed by 38 people (28.4%) treated by 10 or fewer therapists, 14 people (10.4%) treated by 20–29 therapists, 10 people (7.5%) treated by 30–39 therapists, and 10 people (7.5%) who did not know.

#### 3.2.3 Wheelchair usage history

As summarized in Table 7, the majority of respondents (96 individuals, 71.6%) reported using manual wheelchairs. In addition, 32 respondents (23.9%) used both manual and powered wheelchairs, while 6 respondents (4.5%) used only powered wheelchairs. Regarding the duration of wheelchair use, 66 respondents (49.3%) had used a wheelchair for 10 years or less, 48 (35.8%) for 11–20 years, 12 (9.0%) for 21–30 years, and 8 (6.0%) for more than 30 years. Daily wheelchair usage time was reported as 6–10 h by 58 respondents (43.3%), 11–15 h by 50 respondents (37.3%), 1–5 h by 16 respondents (11.9%), and 16–20 h by 10 respondents (7.5%). In terms of self-assessed wheelchair skill level, 64 respondents (47.8%) considered themselves skilled, followed by 38 (28.4%) who considered themselves very skilled, 22 (16.4%) as average, and 10 (7.5%) as unskilled (Table 7).

**Table 7:** Wheelchair usage characteristics: type, duration, daily usage, and self-assessed skill level (*N* = 134).

Division	Contents	Frequency (*N*, %)
Type of wheelchair used	Manual	96 (71.6)
	Powered	6 (4.5)
	Both manual and powered	32 (23.9)
Duration of wheelchair use (years)	≤10 years	66 (49.3)
	11–20 years	48 (35.8)
	21–30 years	12 (9.0)
	31+ years	8 (6.0)
Daily wheelchair use (hours)	1–5	16 (11.9)
	6–10	58 (43.3)
	11–15	50 (37.3)
	16–20	10 (7.5)
Self-assessed wheelchair skill level	Very skilled	38 (28.4)
	Skilled	64 (47.8)
	Average	22 (16.4)
	Unskilled	10 (7.5)

#### 3.2.4 Experience with wheelchair skill training, evaluation, and program awareness

As shown in Table 8, 64 respondents (47.8%) reported having learned wheelchair operation techniques from experts, whereas 70 respondents (52.2%) had not received such training. Among those who had training experience, the majority learned from therapists (58 respondents, 43.3%) and fellow wheelchair users (46 respondents, 34.3%). A smaller number reported learning through self-instruction (4 respondents, 3.0%), other sources such as printed materials or informal instruction (24 respondents, 17.9%), and the internet (2 respondents, 1.5%). Regarding feedback or evaluation of their own wheelchair skills, 28 respondents (20.9%) indicated that they had received such evaluation, while 106 respondents (79.1%) had not. Evaluations were most commonly provided by fellow users (52 respondents, 38.8%), followed by therapists (26 respondents, 19.4%), and other sources (56 respondents, 41.8%). With respect to awareness of training and evaluation tools or manuals related to wheelchair skills, 24 respondents (17.9%) were aware of their existence, while 110 (82.1%) reported having no such knowledge (Table 8).

**Table 8:** Experiences in wheelchair skill training, evaluation, and program awareness (*N* = 134).

Division	Contents	Frequency (*N*, %)
Learned wheelchair skills from experts	Yes	64 (47.8)
	No	70 (52.2)
Wheelchair skill educator	Therapist	58 (43.3)
	Fellow users	46 (34.3)
	Self	4 (3.0)
	etc.	24 (17.9)
	Internet	2 (1.5)
Received evaluation of personal wheelchair skills	Yes	28 (20.9)
	No	106 (79.1)
Wheelchair skill evaluator	Therapist	26 (19.4)
	Fellow users	52 (38.8)
	etc.	56 (41.8)
Awareness of wheelchair skill programs (WSP, WST)	Yes	24 (17.9)
	No	110 (82.1)

#### 3.2.5 Perceived usefulness and participation willingness regarding wheelchair skill training and evaluation tools

As shown in Table 9, in response to the usefulness of promoting the dissemination of the Korean version of the wheelchair skill program (including training and testing tools), 56 respondents (41.8%) rated it as very useful, 64 (47.8%) as useful, 12 (9.0%) as average, and 2 (1.5%) as not useful. Regarding the opinion that even wheelchair users with over 10 years of experience may have improper operation skills, 50 respondents (37.3%) strongly agreed, 66 (49.3%) agreed, 12 (9.0%) were neutral, and 6 (4.5%) disagreed. When asked about their willingness to participate in a wheelchair skill training and education program, 96 respondents (71.6%) indicated “yes,” while 38 (28.4%) responded “no.” In terms of the preferred format for such a program, 52 respondents (38.3%) preferred an internet or mobile-based format, 48 (35.8%) preferred a program conducted by clinical experts at medical institutions, 14 (10.4%) were interested in research participation, and 20 (14.9%) indicated no interest in participating. Regarding reasons for difficulty in participating in such programs, 46 respondents (34.3%) cited difficulty in making time, 30 (22.4%) mentioned cost issues, 24 (17.9%) responded lack of necessity, 12 (9.0%) identified trust issues, and 22 (16.4%) selected other reasons. As for the perceived usefulness of specialized websites or mobile applications for wheelchair skill training and evaluation, 44 respondents (32.8%) found them very useful, 66 (49.3%) useful, 20 (14.9%) average, and 4 (3.0%) not useful. Regarding similar digital tools for clinical experts, 62 respondents (46.3%) found them very useful, 56 (41.8%) useful, 14 (10.4%) average, and 2 (1.5%) not useful.

**Table 9:** Perceived usefulness and preferred formats for wheelchair skill training, evaluation, and education programs (*N* = 134).

Division	Description	Frequency (*N*, %)
Perceived usefulness of disseminating the Korean wheelchair skill program	Very useful	56 (41.8)
	Useful	64 (47.8)
	Average	12 (9.0)
	Not useful	2 (1.5)
Agreement with the statement: long-term users may have improper skills	Strongly agree	50 (37.3)
	Agree	66 (49.3)
	Neutral	12 (9.0)
	Disagree	6 (4.5)
Willingness to participate in a wheelchair skill training and education program	Yes	96 (71.6)
	No	38 (28.4)
Preferred format of training and education program	A program conducted by clinical experts at medical institutions	48 (35.8)
	Internet or mobile form	52 (38.3)
	Research participation	14 (10.4)
	Not interested in participating	20 (14.9)
Reasons for difficulty in participating in the program	Difficulty in making time	46 (34.3)
	Cost issues	30 (22.4)
	Lack of necessity	24 (17.9)
	Trust issues	12 (9.0)
	Others	22 (16.4)
Usefulness of digital training tools for wheelchair users	Very useful	44 (32.8)
	Useful	66 (49.3)
	Average	20 (14.9)
	Not useful	4 (3.0)
Usefulness of digital training tools for clinical professionals	Very useful	62 (46.3)
	Useful	56 (41.8)
	Average	14 (10.4)
	Not useful	2 (1.5)

#### 3.2.6 Awareness of existing wheelchair skill assessment tools

When asked about their awareness of the original Wheelchair Skills Program (WSP), Wheelchair Skills Test (WST), and Transfer Assessment Instrument (TAI), none of the 67 wheelchair user respondents reported familiarity with any of the tools. Among the 60 clinical expert respondents, only one individual indicated awareness of all three tools.

## 4 Discussion

### 4.1 Awareness and utilization of wheelchair skills programs in Korea

This study surveyed both physical therapists and wheelchair users to evaluate the awareness, usage, and necessity of wheelchair skill training and evaluation programs in Korea. Although the original Wheelchair Skills Program (WSP) and Wheelchair Skills Test (WST) have been shown to be effective in improving wheelchair mobility and safety in Western countries (13, 21), their adoption in Korea remains minimal. For instance, only 1.7% of clinical professionals surveyed were aware of the Wheelchair Skills Test Questionnaire (WST-Q), and 95% had never used any training or evaluation tools related to wheelchair skills. Among wheelchair users, 82.1% reported no awareness of such tools. These findings highlight a significant knowledge gap across both key groups.

Although the Korean Wheelchair Skills Program (K-WSP) was introduced in 2019 to address the specific environmental and cultural challenges in Korea—such as narrow hallways, uneven pavements, and floor-based living—its dissemination remains limited. Most therapists and users continue to be unaware of the program, and few clinical institutions incorporate it into their rehabilitation routines.

One major implementation barrier identified is the contextual mismatch of Western programs, which often assume infrastructure like wide corridors and accessible public transport. In contrast, Korean users face environments where low-level transfers, stair navigation, and tight indoor spaces are more common. Therefore, conventional WSP content may not sufficiently address these needs. Another critical barrier is the heavy workload of Korean physical therapists. According to this study, 47.8% of therapists reported treating 21–30 patients per day, with 13.5% handling more than 30. These numbers exceed national recommendations and leave little time for individualized wheelchair skill training.

These findings not only confirm a critical lack of awareness and implementation of wheelchair skill programs in Korea, but also underscore the urgent need for locally adapted education and evaluation frameworks that reflect the realities of Korean users and clinical settings.

### 4.2 Comparative analysis between clinical professionals and wheelchair users

**Figure 1:**
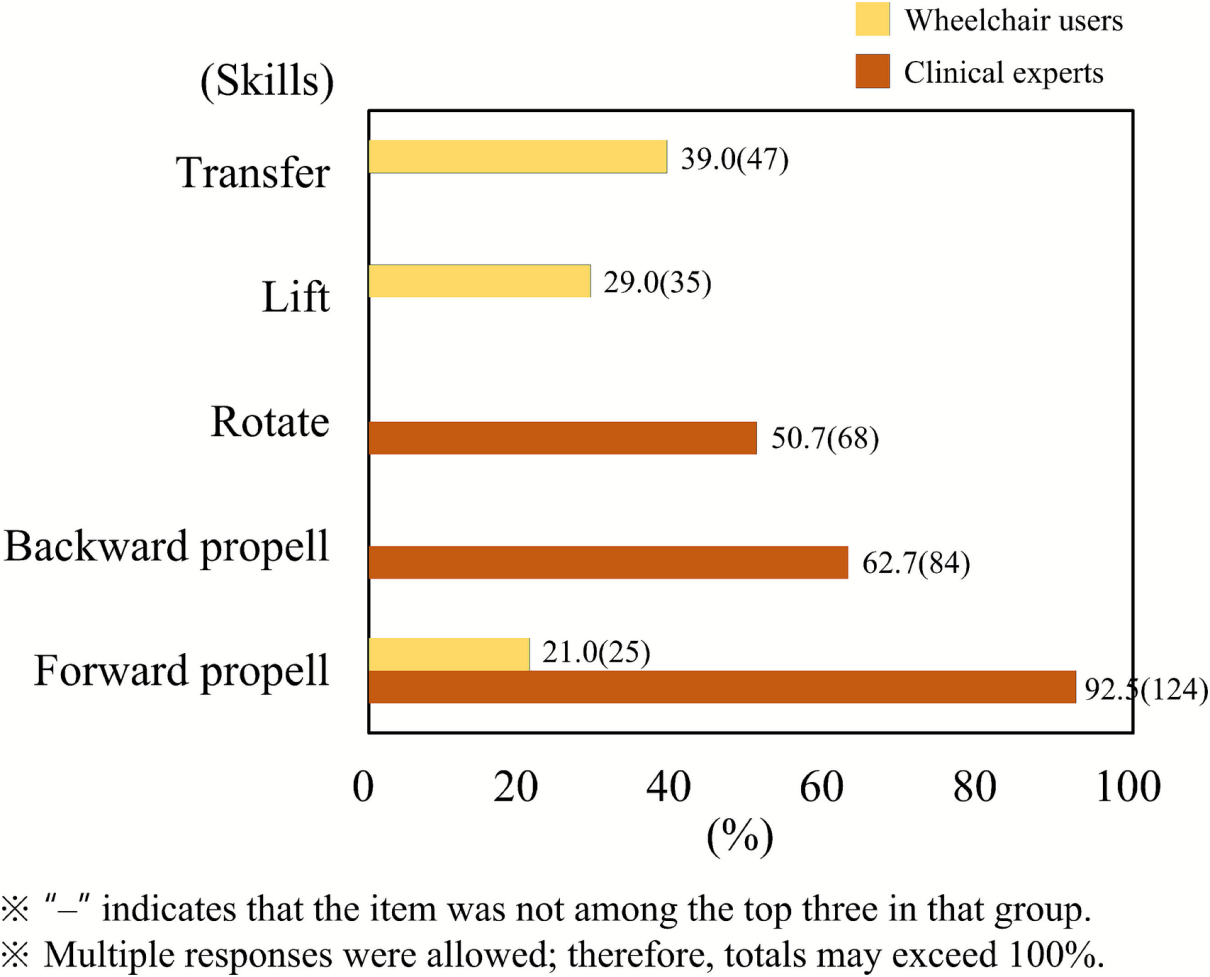
Frequently taught skills by clinical professionals versus frequently performed skills by wheelchair users. All skill categories showed statistically significant group differences (all *χ*^2^ tests *p* < 0.001). Clinical professionals most taught “transfer,” “body lift,” and “forward propulsion,” while users most frequently performed “forward propulsion,” “reverse propulsion,” and “turning.” This discrepancy demonstrates that clinical training does not fully reflect real-world usage.

This study revealed statistically significant differences between clinical professionals and wheelchair users regarding their experiences, priorities, and perceptions of wheelchair skill education. Clinical professionals, all physical therapists with predominantly less than 5 years of experience, reported treating an average of around 10 wheelchair users per day. The three most frequently taught skills were “transfer,” “body lift,” and “forward propulsion.” In contrast, wheelchair users most frequently performed “forward propulsion,” followed by “reverse propulsion” and “turning” (Figure 1). Chi-square tests confirmed that the differences across all listed skills were highly significant (all *p* < 0.001), with large effect sizes (*φ* = 0.42–0.73), indicating that clinical training focuses more on basic or safety-oriented tasks, while users encounter more complex maneuvers in daily contexts, such as reversing or turning in confined spaces. These results highlight the necessity of expanding clinical skill training content to better match real-world mobility demands.

**Figure 2:**
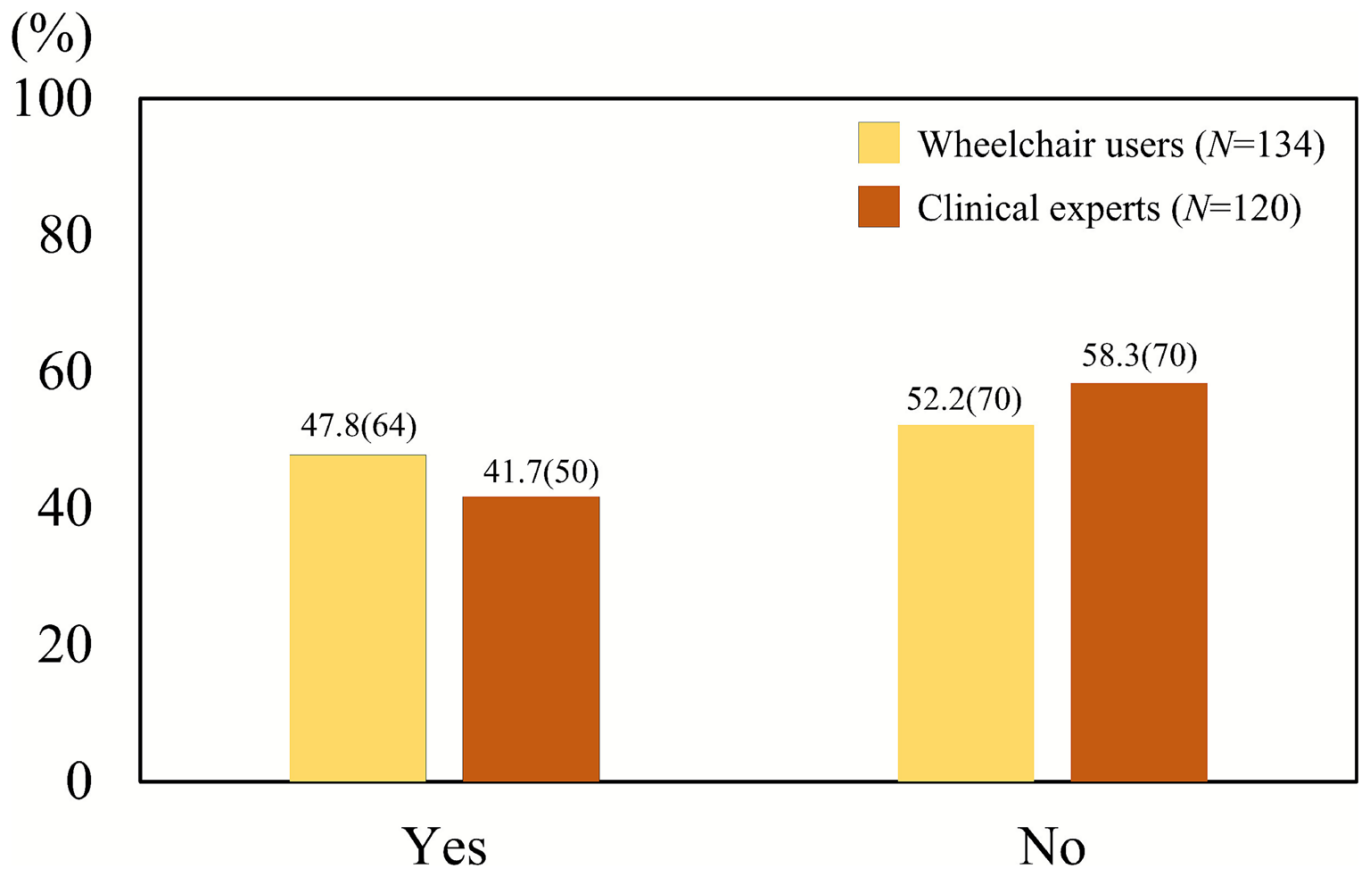
Experience of receiving wheelchair skill education from experts. A majority of both clinical professionals (58.3%) and wheelchair users (52.2%) reported not having received formal education on wheelchair operation techniques. The difference between groups was not statistically significant (*χ*^2^ = 0.95, *p* = 0.33).

A lack of formal education on wheelchair skills was also evident in both groups. More than half of clinical professionals (58.3%) and wheelchair users (52.2%) stated that they had not received any formal instruction on wheelchair operation techniques from qualified professionals (Figure 2). Statistical analysis, however, showed no significant difference between the two groups (*χ*^2^ = 0.95, *p* = 0.33, *φ* = 0.06), suggesting that this lack of training is a pervasive issue across both providers and users.

**Figure 3:**
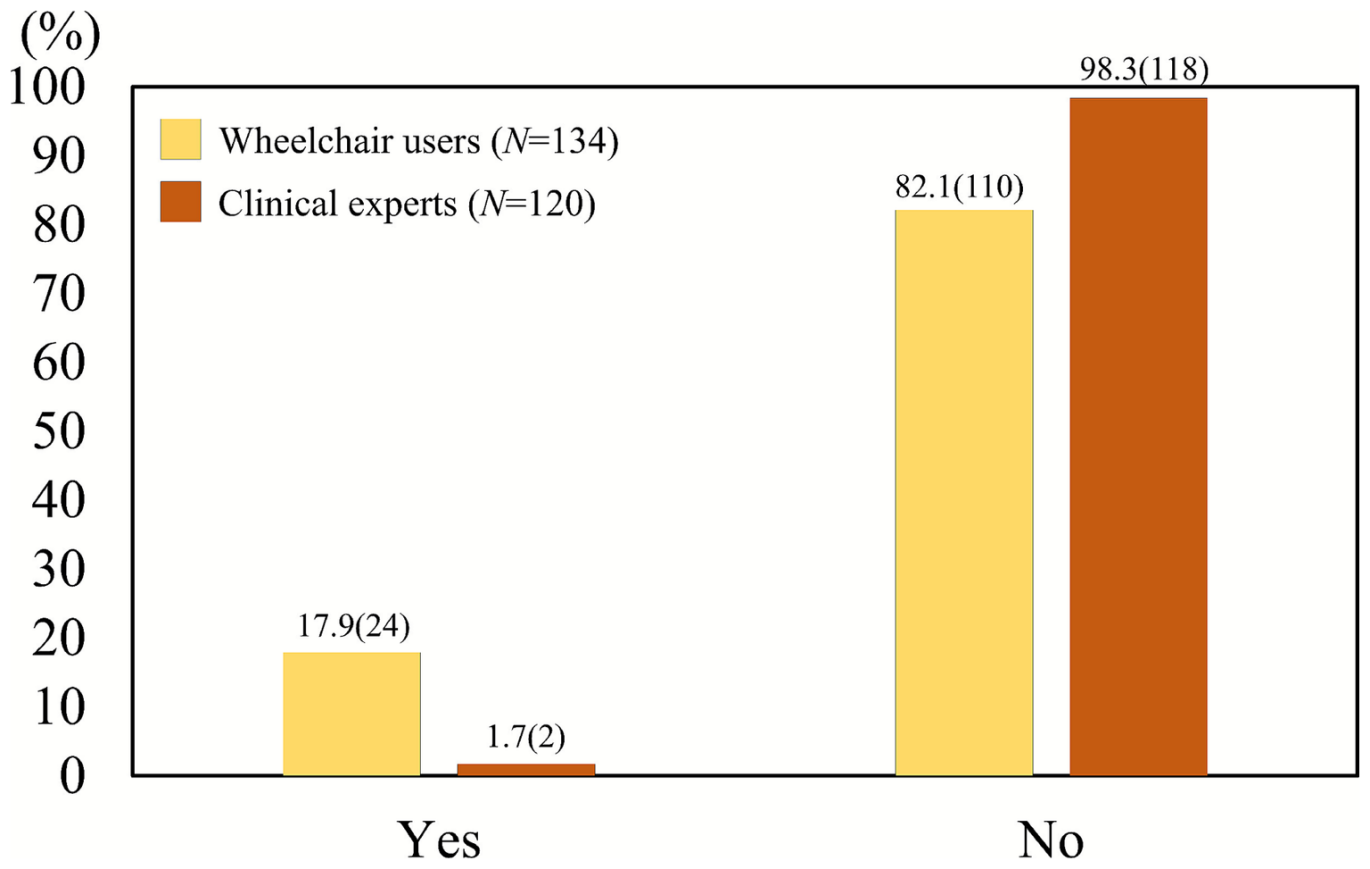
Awareness of wheelchair skill training and evaluation tools (e.g., WSP, WST, manuals). Awareness was extremely low in both groups, but the difference between users (17.9% aware) and clinicians (1.7% aware) was significant (*χ*^2^ = 18.18, *p* < 0.001; Fisher’s exact *p* < 0.0001).

In terms of awareness of training and evaluation tools, such as the Wheelchair Skills Program (WSP) or Wheelchair Skills Test (WST), the gap was striking: 98.3% of clinical professionals and 82.1% of wheelchair users reported no awareness of such resources (Figure 3). This difference was statistically significant (*χ*^2^ = 18.18, *p* < 0.001; Fisher’s exact *p* < 0.0001, *φ* = 0.27), indicating that professionals were even less aware of standardized tools than users. These findings reveal critical gaps not only in user education but also in professional training systems.

**Figure 4:**
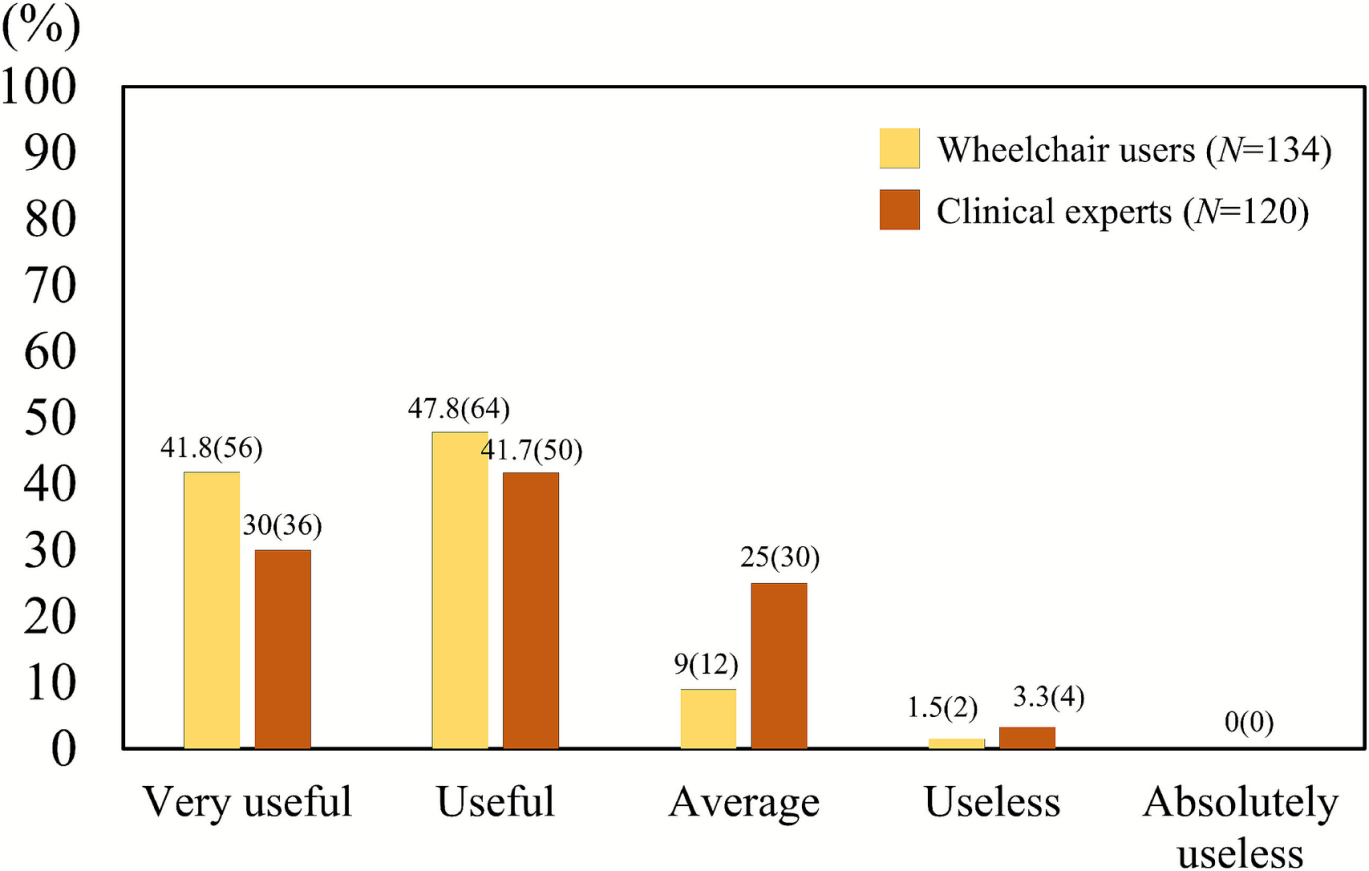
Perceived usefulness of a Korean-style wheelchair skill training and evaluation program. Both groups showed high support (71.7% of clinicians, 89.6% of users), but users were significantly more likely to rate the program as “very useful” (*χ*^2^ = 15.26, *p* = 0.0016).

Despite these gaps, both groups agreed on the usefulness of developing a Korean-style wheelchair skill training and testing program. A large majority of clinical professionals (71.7%) and an even higher proportion of users (89.6%) rated such a program as useful or very useful (Figure 4). Chi-square analysis confirmed a significant difference in response distributions (*χ*^2^ = 15.26, *p* = 0.0016, *V* = 0.25), with users more strongly endorsing the program as “very useful.” Thus, while consensus exists on the need for such a program, users expressed stronger enthusiasm.

**Figure 5:**
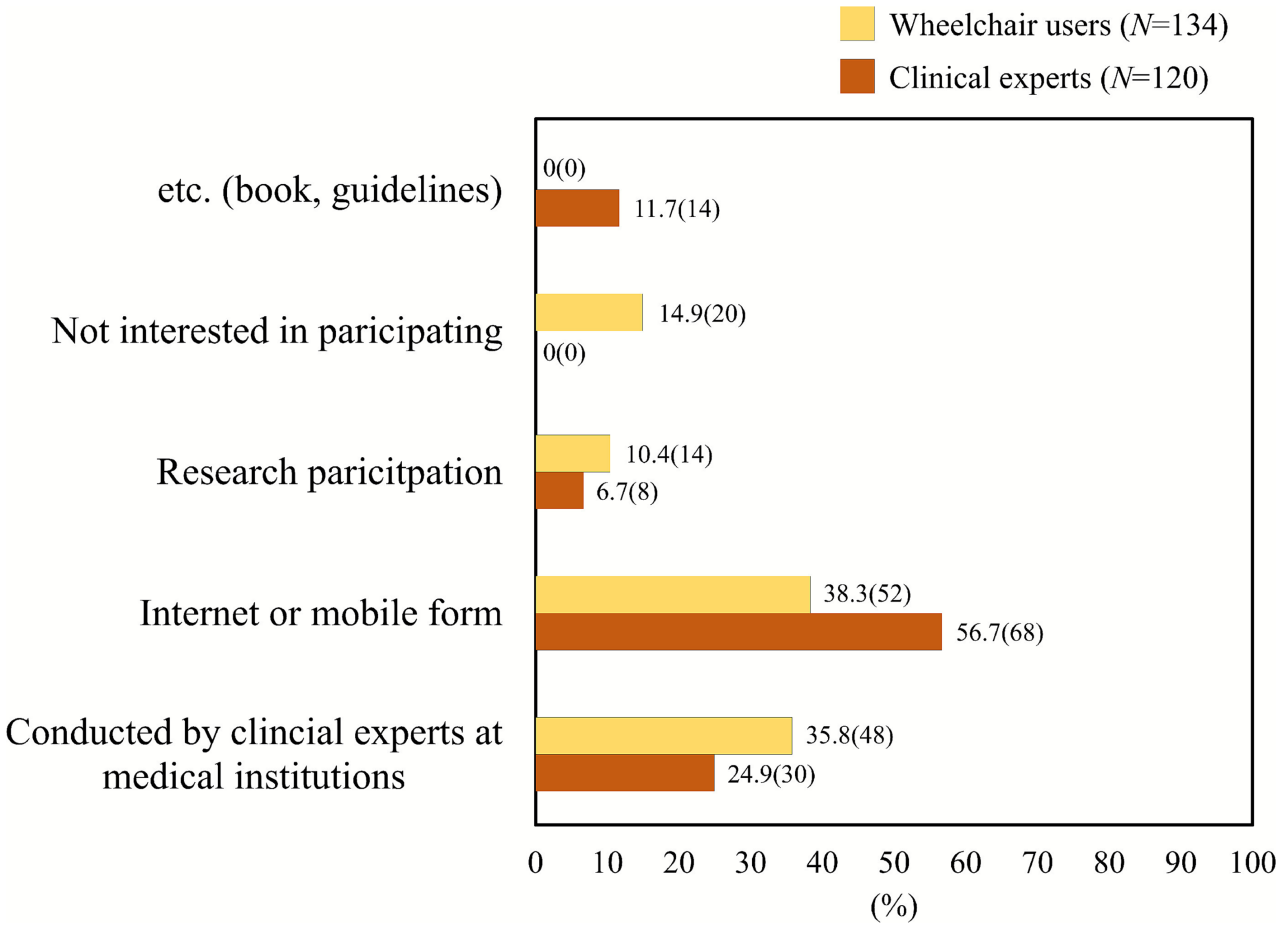
Preferred delivery format of the Korean-style wheelchair skill program. Distributional differences were highly significant (*χ*^2^ = 43.93, *p* < 0.0001). Clinical professionals preferred online/mobile-based formats (56.7%), while users showed nearly equal preference for mobile applications (38.3%) and in-person instruction from clinical professionals (35.8%).

Finally, preferences regarding program delivery formats differed considerably. Clinical professionals favored digital formats (56.7% online or mobile), likely due to time constraints in clinical practice, whereas users expressed nearly equal preference for mobile applications (38.3%) and direct instruction from professionals in medical institutions (35.8%) (Figure 5). The group difference in distribution was highly significant (*χ*^2^ = 43.93, *p* < 0.0001, *V* = 0.42), underscoring that while professionals prioritize convenience, users still value face-to-face expert guidance.

These insights emphasize the need for a clearer, user-centered program design that directly reflects real-world demands. Future development should focus on integrating provider feasibility with user needs, ensuring that both perspectives are addressed. For example, training content should be expanded to include reverse and turning maneuvers, and delivery methods should offer flexible options such as digital and face-to-face formats. This approach would reduce gaps between what providers offer and what users require, ultimately improving program participation and effectiveness.

It is also important to note the scope and limitations of this study. Clinical professionals were limited to physical therapists, and the user group was composed solely of individuals with spinal cord injury (SCI), which reflects the predominant wheelchair user demographic in South Korea. Future studies should include other user populations, such as individuals with neuromuscular or musculoskeletal disorders (e.g., muscular dystrophy), and incorporate the perspectives of occupational therapists and other rehabilitation professionals who play a key role in wheelchair skill education.

Finally, although data collection was supported by several national organizations, the response rate was lower than expected. The extended survey period required to meet the target sample size illustrates the practical challenges of engaging a broad and diverse participant base, especially in studies involving individuals with mobility impairments. This highlights the need for improved outreach strategies and stronger institutional support in future research and program implementation.

### 4.3 Barriers to implementation in the Korean healthcare context

The findings of this study highlight several systemic barriers that hinder the implementation of structured wheelchair skills education in the Korean healthcare system. Chief among these is the high patient load assigned to physical therapists. According to the survey, 47.8% of clinical professionals treat between 21 and 30 patients per day, and 13.5% treat more than 30. These figures far exceed the recommended caseload suggested by the National Health Insurance Service and limit the time available for individualized wheelchair skill instruction.

This workforce strain is further compounded by the relatively low number of physical therapists per capita in Korea. According to OECD statistics, Korea has only 0.84 physical therapists per 1,000 population, which is significantly lower than the OECD average of 1.09 (22). This shortage of rehabilitation professionals increases the clinical burden and leaves little flexibility for the adoption of new training programs such as the Wheelchair Skills Program (WSP) or the Korean-adapted version (K-WSP).

In addition to quantitative shortages, the survey also revealed qualitative barriers. Many therapists lack awareness or formal training in wheelchair skill education, and most clinical settings lack the institutional framework or equipment necessary to integrate these programs into routine practice. For instance, 98.3% of professionals surveyed reported no awareness of standardized manuals or evaluation tools, and only 5% had any experience using training or testing materials.

Furthermore, while clinical professionals expressed interest in digital delivery methods for educational content, their ability to implement such tools is constrained by limited institutional support, time, and digital infrastructure. Without policy-level backing, financial incentives, or mandatory guidelines, the integration of wheelchair skill education into the national rehabilitation curriculum remains challenging.

Taken together, these findings indicate that both human resource limitations and systemic infrastructure gaps are significant obstacles to the implementation of wheelchair skill programs in Korea. Addressing these barriers will require multi-level interventions, including increased workforce capacity, enhanced therapist training, and policy-driven support for standardized education in rehabilitation practice.

### 4.4 Recommendations for future development and dissemination

Based on the findings of this study, several strategic directions are proposed to facilitate the effective development and dissemination of wheelchair skills training and evaluation programs within the Korean rehabilitation context.

First, it is recommended that established programs such as the Wheelchair Skills Program (WSP) and Wheelchair Skills Test (WST) be systematically incorporated into the formal educational curricula for physical therapy and occupational therapy students. Early exposure during professional training may enhance future clinicians’ competency and promote greater familiarity with standardized wheelchair skill education.

Second, continuing professional development programs should be expanded to include structured training in WSP and WST for currently practicing rehabilitation professionals. Given the widespread lack of awareness and practical experience observed among clinical experts in this study, targeted in-service education is essential for bridging knowledge gaps and enhancing clinical implementation capacity.

Third, policy-level support must be strengthened to enable the nationwide adoption of wheelchair skills training programs. National health authorities and relevant professional bodies should consider incorporating these programs into official rehabilitation guidelines. Additionally, institutional resources such as funding, equipment, and staffing support must be allocated to facilitate practical implementation in clinical and community-based settings.

Fourth, the use of digital platforms, including web-based modules and mobile applications, should be promoted to improve accessibility, cost-effectiveness, and scalability. Digital delivery formats may help overcome the time and space constraints frequently encountered by both clinicians and users, thereby increasing program reach and user engagement.

Collectively, these strategies are expected to support the development of a sustainable and contextually appropriate wheelchair skills training framework in Korea. A coordinated approach involving educational institutions, clinical facilities, and policy-making entities is essential to ensure the successful integration of such programs into routine rehabilitation practices, ultimately contributing to safer and more independent mobility among wheelchair users.

## 5 Conclusion

This research identified low awareness and minimal use of wheelchair skills training materials among both wheelchair users and healthcare professionals in Korea. Although programs such as the WSP and WST have shown effectiveness internationally, they remain rarely used in Korean clinical or community settings. The findings also showed differences between therapists and wheelchair users regarding which skills they considered important—therapists focused on basic or safety-oriented techniques, whereas users expressed greater needs for skills such as reverse propulsion and turning.

These results indicate a clear gap between current clinical practice and the mobility demands experienced by wheelchair users. Improving awareness of wheelchair skills training and developing resources that fit the Korean context will be essential. The findings should be interpreted cautiously due to the limited participant groups and the use of convenience sampling. Future studies should include a broader range of users and rehabilitation professionals and evaluate structured wheelchair skills programs in actual clinical and community environments.

## Data Availability

The datasets generated for this study are available on request to the corresponding author.
